# Channel Geometry Controls on Chemical Behavior in Rivers: Insights from a Comparative Field Study

**DOI:** 10.1021/acsestwater.4c01203

**Published:** 2025-09-09

**Authors:** Robert A. Newbould, D. Mark Powell, Juliet Hodges, Alexandre Teixeira, Ian Guymer, Michael J. Whelan

**Affiliations:** ‡ School of Geography, Geology and the Environment, 4488 University of Leicester, Leicester LE1 7RH, U.K.; § Safety, Environmental and Regulatory Science, Unilever, 3099 Colworth Science Park, Sharnbrook MK44 1LQ, U.K.; ⊥ School of Mechanical, Aerospace and Civil Engineering, 7315 The University of Sheffield, Sheffield S1 3JD, U.K.

**Keywords:** wastewater, dye tracing, geomorphology, chemical exposure, biodegradation, nitrification

## Abstract

Microbially mediated transformations, such as nitrification and biodegradation, play a crucial role in removing pollutants from rivers. Although in-stream removal rate coefficients are often assumed to be spatially and temporally constant, they are likely affected by the channel shape and size because these factors control contact between the water column and fixed biofilms. Here, we test the hypothesis that transformation rate constants are inversely proportional to the hydraulic radius (*R*: ratio of the channel cross-sectional area to wetted perimeter) in dye tracing experiments conducted in two U.K. rivers with contrasting morphologies: (1) the River Maun (shallow: mean bankfull *R* = 1.25 m) and (2) the River Calder (deep: mean bankfull *R* = 3 m). In each case, a slug of rhodamine WT was injected upstream of a wastewater outfall, and samples were collected downstream, staggered by the rhodamine travel time. Rate constants were derived for sucralose, ammonium, caffeine, and linear alkylbenzenesulfonate. Sucralose (persistent, hydrophilic, and exclusively of wastewater origin) was used as a conservative tracer to adjust model fits for dilution. Higher rate coefficients were observed for all biotransformed pollutants in the Maun compared to the Calder, supporting the hypothesis and highlighting the need to consider geomorphology in models of chemical behavior.

## Graphical abstract

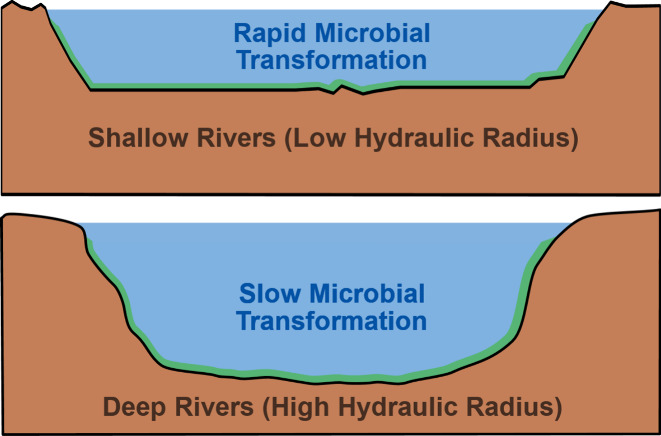

## Introduction

The emission of pollutants into riverine environments poses a potential risk for aquatic organisms, and for human health, if river water is used for water supply. Wastewater represents an important pollutant source. Although wastewater treatment is often able to remove a high proportion of many pollutants, residual concentrations in river water downstream of sewage treatment plants (STPs) can still pose substantial ecotoxicological risks.^1,2^ Pollutants associated with wastewater include organic compounds, such as those found in pharmaceuticals and personal care products, inorganic contaminants, such as ammonia, nitrite, and heavy metals, and nonspecific degradable organic matter, which can impose a biochemical oxygen demand on receiving waters.^1^ To quantify exposures of wildlife and humans to these contaminants for environmental risk assessments, we need to understand how they dissipate in receiving environments under different conditions. In rivers, a number of mechanisms can contribute to pollutant removal, including microbially mediated transformations (e.g., nitrification and biodegradation), sorption to sediment, volatilization, and photodegradation.^3–5^ The relative importance of these mechanisms depends on (1) intrinsic properties of individual chemicals (e.g., partition coefficients and chemical structure) and (2) environmental conditions, such as temperature, pH, dissolved oxygen concentration, and short-wave radiation flux density.^5^

Microbially mediated transformations are a dominant removal mechanism for many wastewater pollutants. These are performed by both suspended organisms and microbes in fixed biofilms, growing on boundary sediment and on the surfaces of vegetation.^3,6–9^ Biofilms are believed to be much more significant for the processing of chemicals in most rivers and streams, compared with suspended organisms, because their biomass is typically much higher and their communities are more diverse.^6,10,11^ Sediments at the sediment–water interface, and in the hyporheic zone, are therefore often termed “bioreactors” due to their high potential to degrade wastewater pollutants.^12–14^

The high importance of biofilms at the sediment–water interface implies that microbially mediated transformations should be affected by contact between the water column and sediment surfaces. This will be controlled to some extent by the channel shape and size.^10,15,16^ Specifically, hydraulic radius (*R*: ratio of the channel cross-sectional area to the wetted perimeter, *P*) can be used as a measure of the volume of streamwater available for processing by a unit area of bed sediment. *R* is often used to describe the efficiency of a channel to transport water and sediment.^17^ In many natural channels, *P* is approximately equal to the channel width (*w*).^17,18^ *R* can then be approximated by the flow depth (*d*).^17,18^ Although local channel characteristics can vary substantially, catchment scale changes tend to be systematic and controlled by river discharge.^18,19^ In most rivers in humid catchments, river discharge typically increases with the drainage area (i.e., with distance downstream). This increase in discharge is usually associated with systematic increases in channel width and depth, as well as a progressive decline in channel gradient.^18,19^ In parallel, there is often an increase in mean velocity associated with an increase in *R* and a systematic decrease in channel roughness due to progressive decreases in the mean sediment caliber.^17–20^ Empirical relationships between channel characteristics and discharge are often referred to as hydraulic geometry equations.^18,19^

We hypothesize that rate constants for microbial transformations (*k*) will be inversely proportional to *R* (i.e., microbial transformations will be quicker in shallow rivers, with low *R*, compared to deep rivers, with high *R*):^6,21^

1 $$k\propto \frac{1}{R}$$

Transformation rates are also expected to be affected by temperature and light penetration, which will both be higher in shallow compared to deep rivers.^22–26^ In addition, increased stream turbulence in shallow rivers is expected to increase vertical mixing and the delivery of contaminants to the streambed for processing, thereby increasing *k*.^27,28^ These broadly applicable and systematic controls have important implications for both (1) exposure to contaminants locally (and associated ecotoxicological risk) and (2) the flux of contaminants to the coastal zone.

These factors have been invoked to explain observations of reduced nitrification rates with increasing depth in rivers and free surface constructed wetlands^29,30^ for denitrification in lakes, reservoirs, and rivers^15,31–34^ and for chemical biodegradation in rivers.^6,10^ Previous work that attributes reduced rates of microbial transformations to differences in depth were conducted under controlled laboratory conditions or by fitting models to monitoring data.^6,10,15,29–34^ However, it is widely recognized that laboratory-derived rate constants often differ from those in the field.^3,10,35^ Furthermore, derivation of rate constants in the field are most reliably obtained using dye tracing techniques in which the travel time of an external tracer is used to guide the timing of sample collection at downstream stations, such that the same parcel of water is sampled.^36,37^ Such studies are rarely conducted, so reliable field-derived rate constants for many contaminants do not exist. In this paper, we derive, for the first time, rate constants for a suite of wastewater contaminants in two rivers with contrasting hydraulic geometries in order to test the hypothesis that the rate constant should be inversely proportional to *R*.

## Methods

### Study Areas

Dye tracing experiments were conducted in two rivers with contrasting channel morphologies: (1) the River Maun, which receives wastewater from Mansfield STP, Nottinghamshire, U.K., and (2) the River Calder, which receives wastewater from Dewsbury (Mitchell Laithes) STP, West Yorkshire, U.K. (Figure 1). The criteria for choosing each study site were (1) the presence of a strong wastewater signal from a wastewater treatment works, with several kilometers of downstream reach before the next significant wastewater outfall, and (2) a significant contrast in the channel geometry characteristics between sites (relatively shallow in the case of the Maun and much deeper for the Calder).

**Figure 1:**
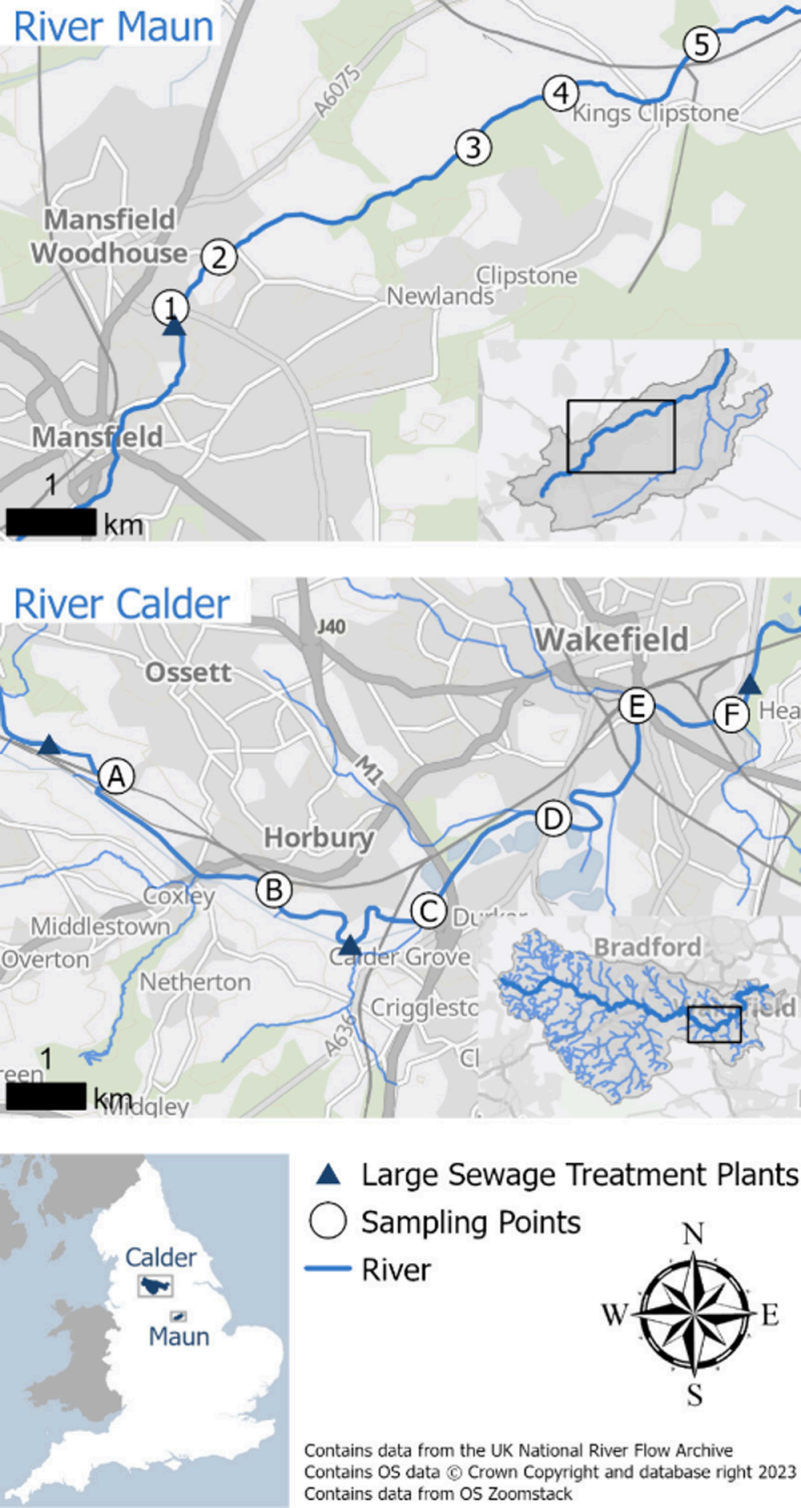
Location of study catchments, sampling points, and major STPs along the sampled reaches of the Rivers Maun and Calder. Data are from the U.K. National River Flow Archive. OS data © Crown Copyright and database right 2023. Data are from OS Zoomstack.

Mansfield STP (53°09′22.6″N 1°10′54.6″W) serves a population of 97000 people.^38^ It discharges into the River Maun, a shallow river which flows through Mansfield before joining the River Idle. The approximate bankfull width and depth in the study reach are 5 and 1.25 m, respectively.^39^ Land cover in the catchment upstream of the effluent discharge point is predominantly urban (63%) with arable farmland (17%) and grassland (14%).^40^ Mean river discharge (*Q*) at the effluent discharge point is 0.457 m^3^ s^–1^ and the catchment area is 28.8 km^2^.^40^ Wastewater effluent makes up a high proportion of flow in the River Maun, with an estimated average dilution factor (DF: ratio of total discharge to effluent discharge) of 4.4.^38^

Dewsbury STP (53°40′20.7″N, 1°36′21.3″W) serves a population of 380000 people^38^ and discharges into the River Calder. The Calder rises in the Pennine Hills and has many tributary inputs before reaching Dewsbury. Downstream of Dewsbury, the Calder flows through Wakefield before joining the River Aire at Castleford. The approximate bankfull width and depth in the study reach are 27 and 3 m, respectively.^39^ Land cover in the Calder catchment upstream of Dewsbury is predominantly grassland (46%) with significant areas of heathland (18%), woodland (15%), and urban land (15%).^41^ The floodplain is heavily urbanized and industrialized. The mean *Q* and catchment area at Dewsbury are 16.3 m^3^ s^–1^ and 697 km^2^, respectively.^41^ The estimated DF of Dewsbury STP at mean flow is 22.^38^ Although this is not as low as for the Maun at Mansfield STP, a previous monitoring exercise indicated that pollutant concentrations could be detected and tracked.^21,42^ It should be noted that there are some potential interactions between the River Calder and the Calder and Hebble canal system in the monitored reach (e.g., via sluice gates to supply water to the canal and weirs to impound water). However, the majority of flow is retained within the main river channel.

In each field experiment, water samples were collected from one point upstream and several points (five for the Maun and six for the Calder; see Table S2) downstream of the main STP effluent outfall over distances of 7.8 and 13.7 km for the Maun and Calder, respectively. There were no other known discharges of municipal wastewater in the monitored reach on the Maun. There was, however, another municipal STP discharging to the Calder downstream of Dewsbury at Horbury (population served, 16000 people; DF, 504).^38^ We calculated that this would have a negligible effect on the concentrations of the contaminants of interest due to high dilution (explored in more detail later).

### Hydraulic Geometry

Although channel dimensions changed continuously with distance downstream in each river, there were fundamental differences in geometry between the two monitored reaches. Three approaches were used to characterize *R*, all of which assume that *R* is approximated by *d* (see the Supporting Information for justification). The first approach estimated *d* using the simple relationship

2 Q=wdv

where *v* is the velocity (m s^–1^).^19^ *Q* and *v* were obtained through dilution gauging (using numerical integrals of dye concentrations at each station)^43^ and solute travel time (see below), respectively, while *w* was measured using satellite imagery.

The second approach utilized gridded estimates of bankfull *d* from the UK Centre for Ecology and Hydrology.^39^ This data product was derived from a hydraulic geometry relationship between *d* and the product of catchment area (*A*, km^2^) and mean annual rainfall (*R*_f_, mm), which is a proxy for *Q*:

3 $$d=0.02643A^{0.202}{R_{f}}^{0.482}$$

This relationship was calibrated against historical survey data, with full details available in Davies et al.^39^ The final approach to estimate *d* involved using stage data from the Environment Agency (environment.data.gov.uk/hydrology). River stage represents the height of a river relative to a fixed point on or near the river bed (local datum). Gauging stations were located between Sites 2 and 3 in the River Maun and at Site B in the River Calder.

### Sample Collection

A Lagrangian sampling approach was employed in which the collection of water samples was staggered to coincide with the solute travel time. This is important because any changes in pollutant concentrations can then be attributed to transformation, losses from or gains to the water column, rather than simply the sampling of different parcels of water.^3,36,37,44^ The fluorescent dye rhodamine WT (Town End, Leeds, U.K.) was used as a conservative tracer to determine the solute travel time (eq S1).^45^ This was introduced as a slug injection (50 mL to the Maun and 1000 mL to the Calder) in the midchannel upstream of the main STP in each river (200 m for the Maun and 2000 m for the Calder). Rhodamine WT was also used to estimate river discharge at each station via dilution gauging (eq S2).^46^

A single dye tracing campaign was conducted in each river reach. Sampling on the River Maun was conducted on August 2, 2023. In the River Calder, dye tracing was conducted overnight on February 19, 2024, and water samples were collected the following day, February 20, 2024, using travel times determined from the dye trace. This approach is acceptable under steady-flow conditions. This was confirmed with the stage data at Site B from the Environment Agency (Figure S2). Although sampling was conducted in different seasons, we accounted for the effects of temperature in our analyses (see below).

Fluorescence was measured at each sampling site using Cyclops-7F submersible fluorimeters (Turner Designs, San Jose, CA). Fluorescence data were recorded with Cyclops-7 loggers (Precision Measurement Engineering, Vista, CA) to enable the calculation of the tracer centroid (center of mass). In the Maun, a hand-held Cyclops-7F was employed to collect water samples around the peak dye concentration, with the aim of capturing samples at the tracer centroid. Water samples were collected in triplicate in 60 mL HDPE plastic bottles and stored on ice for up to 24 h before being frozen, prior to analyses. HDPE plastic bottles were new and rinsed with river water prior to sample collection. *In situ* water quality parameters were also measured, including temperature and pH (measured with Electronic Temperature Instruments 8100 Plus pH meter, Worthing, U.K.), dissolved oxygen (DO; measured with a YSI ProODO DO meter, Yellow Springs, OH) and electrical conductivity (EC; measured with Mettler Toledo FiveGo F3 EC meter, Columbus, OH).

Note that samples were collected at Sites C and E on the Calder (Figure 1), but no fluorometers were installed at these stations. Instead, sampling times were estimated from solute travel times using linear interpolation between Sites B and D (for Site C) and between Sites D and F (for Site E).

### Sample Analyses

Water samples were analyzed for a suite of chemicals typically found at detectable concentrations in wastewater with a range of reported transformation profiles and expected rates: sucralose (persistent), ammonium (NH_4_^+^; oxidized to nitrite and then nitrate through nitrification), caffeine (rapidly biodegradable by a range of heterotrophic microorganisms^47^), and linear alkylbenzenesulfonate (LAS; biodegradable by a range of heterotrophic microorganisms^36,37^ ). LAS is a multiconstituent substance composed of alkyl chain homologues of varying length (C_10_–C_12_) and positional isomers. For our analyses, the sum of all LAS homologues was used. All samples were filtered through 0.45 μm PTFE syringe filters prior to any analyses. NH_4_^+^ concentrations were determined colorimetrically with a method equivalent to ISO 15923-1 using a SEAL AQ2 discrete analyzer. The ammonium sulfate standard (molecular biology grade, ≥99%) used was supplied by Sigma-Aldrich (Gillingham, U.K.). All other chemicals were ACS reagent grade (≥95%) or better.

Caffeine, LAS, and sucralose concentrations were determined using liquid chromatography with tandem mass spectrometry and electrospray ionization (Agilent 1290 series LC-MS/MS system with 6495 triple quadrupole mass spectrometer and electrospray ionization source). Analyses were conducted over two separate runs: one in positive-ion mode for caffeine and one in negative-ion mode for LAS and sucralose. Caffeine (≥99%) and sucralose (≥98%) standards were obtained from Sigma-Aldrich. The dodecylbenzenesulfonic acid standard (LAS; 97.3%) was obtained from Cepsa (Madrid, Spain). Caffeine-^13^C_6_ (98.2%; Sigma-Aldrich) was used as an internal standard for positive-ion mode analyses. Sodium dodecyl-d25-sulfate (98.3%; Sigma-Aldrich) was used as the internal standard for negative-ion mode. Limits of quantification (LOQs) were 0.1 μg L^–1^ for caffeine, 0.5 μg L^–1^ for sucralose, 0.9 μg L^–1^ for C_10_ LAS, 2.2 μg L^–1^ for C_11_ LAS, 1.6 μg L^–1^ for C_12_ LAS, and 0.01 mg N L^–1^ for ammoniacal N. Further information about LC-MS/MS conditions, as well as information on method development and validation, are provided as Supporting Information.

### Curve Fitting

We assume that the dominant transformation mechanism for all contaminants investigated is microbially mediated, based on a wide range of literature, although we do recognize that other processes operate. For example, NH_4_^+^ can be lost as un-ionized ammonia to the atmosphere via volatilization and immobilized via plant uptake and net microbial assimilation. In all cases, first-order kinetics was assumed. To fit first-order transformation rate coefficients (*k*) while accounting for downstream dilution, the following equation is typically used:

4 $$C_{t}=\frac{Q_{0}}{Q_{t}}C_{0}\;\exp\left(-kt\right)$$

where *C*_0_ and *Q*_0_ are the concentration and discharge immediately after mixing downstream of the main STP input and *C*_*t*_ and *Q*_*t*_ are the concentration and discharge at time *t* (coinciding with the tracer centroid), respectively. We considered three different ways of estimating dilution (*Q*_0_/*Q*_*t*_): (1) flow accumulation between gauging stations, (2) reductions in the numerical integral of dye concentrations at each station (dilution gauging), and (3) chemical benchmarking using the sucralose concentration at each station. The paucity of gauging stations in each river reduced the practicality of using gauged data. Dilution gauging was performed, but potential abstractions from the River Calder introduced potential errors in estimates of *Q*_*t*_. Benchmarking involves measuring the relative behavior of different chemicals (one of which has known properties and the other unknown), rather than their absolute values.^48^ Here, dilution at each sampling site was accounted for using the ratio of the sucralose concentration at the sampling site to the sucralose concentration immediately downstream of the main STP on each river, after mixing:

5 $$C_{t,\mathrm{DEG}}=\frac{C_{t,\mathrm{PER}}}{C_{0,\mathrm{PER}}}C_{0,\mathrm{DEG}}\;\exp\left(-kt\right)$$

where the subscripts PER and DEG refer to persistent (sucralose) and degradable (NH_4_^+^, caffeine, LAS) compounds, respectively. Sucralose (CAS: 56038-13-2) is an artificial sweetener that is widely used in food products and that is now commonly detected in wastewater.^49,50^ It is hydrophilic (estimated log *K*_OW_ = −1)^51^ and has been shown to be very persistent to biodegradation in both wastewater treatment and in receiving water bodies,^49,52,53^ making it an ideal benchmarking contaminant for this study. If we assume that it is perfectly persistent with zero net sorption (reasonable for a steady-state emission) reductions in sucralose concentrations will be proportional to dilution resulting from hillslope and groundwater contributions to flow accretion. This approach is similar to that previously employed using boron as a persistent tracer.^37^ Boron used to be a common ingredient in laundry detergents, but its use has decreased significantly in recent years, making it impractical as a benchmarking tracer.^54^ *k* was fitted by iterative optimization to reduce the root-mean-square error (RMSE) between observed and modeled concentrations using the generalized reduced gradient algorithm.^55^

### Temperature Correction

To account for the effect of temperature on nitrification and biodegradation, fitted rate constants were normalized to a reference temperature, *T*_r_ (K), using the Arrhenius equation:^56,57^

6 $$k\left(T_{r}\right)=\frac{k\left(T_{e}\right)}{\exp\left[\frac{E_{a}}{R_{g}}\left(\frac{1}{T_{r}}-\frac{1}{T_{e}}\right)\right]}$$

where *k*(*T*_r_) is the rate constant (h^–1^) at the reference temperature, *k*(*T*_e_) is the rate constant (h^–1^) at the environmental temperature, *T*_e_ (K), *R*_g_ is the universal gas constant (8.3145 J mol^–1^ K^–1^), and *E*_a_ is the activation energy. An activation energy for nitrification of 162 kJ mol^–1^ has previously been reported in riverbed sediment cores.^58,59^ In the absence of substance-specific *E*_a_ values for biodegradation, a generic value of 65.4 kJ mol^–1^ was used, as recommended by REACH regulation (EC 1907/2006).^60^ This corresponds to the median value of *E*_a_ data available for pesticides, which were measured in soil.^61^ There is some doubt about the universal application of the Arrhenius equation to normalize *k*(*T*_r_). For example, Tian et al.^9^ measured the biodegradation rates of 96 compounds during different seasons and found deviation from the Arrhenius equation for most of the studied compounds. However, this equation remains the most widely accepted method for temperature correction and has been incorporated into regulatory practice in Europe for both pesticides^62^ and general chemicals.^60^

## Results

### Hydraulic Geometry

To characterize *R* in the monitored river reaches, three approaches were used, each of which assume that *R* is approximated by *d* (Table 1). Estimates of *d* ranged from 0.34 to 1.25 m in the Maun and from 0.85 to 1.45 m in the Calder. Predictably, the estimates of *d* from UK CEH were the highest, as this method estimated bankfull *d* rather than *d* on the day sampling was conducted. The ratio of *d*_Maun_ to *d*_Calder_ ranges from 0.23 to 0.42.

**Table 1:** Estimates of *R*, Assuming That *R* Can Be Approximated by *d*

approach	Maun (m)	Calder (m)	*d*_Maun_:*d*_Calder_
hydraulic geometry depth	0.34	1.45	0.23
Environment Agency stage	0.34	0.85	0.40
UK CEH bankfull depth	1.25	3	0.42

### Solute Travel Time

Total solute travel times for the Rivers Maun (Sites 1–5) and Calder (Sites A–F) were 6.8 and 8 h, respectively (Figure 2).

**Figure 2:**
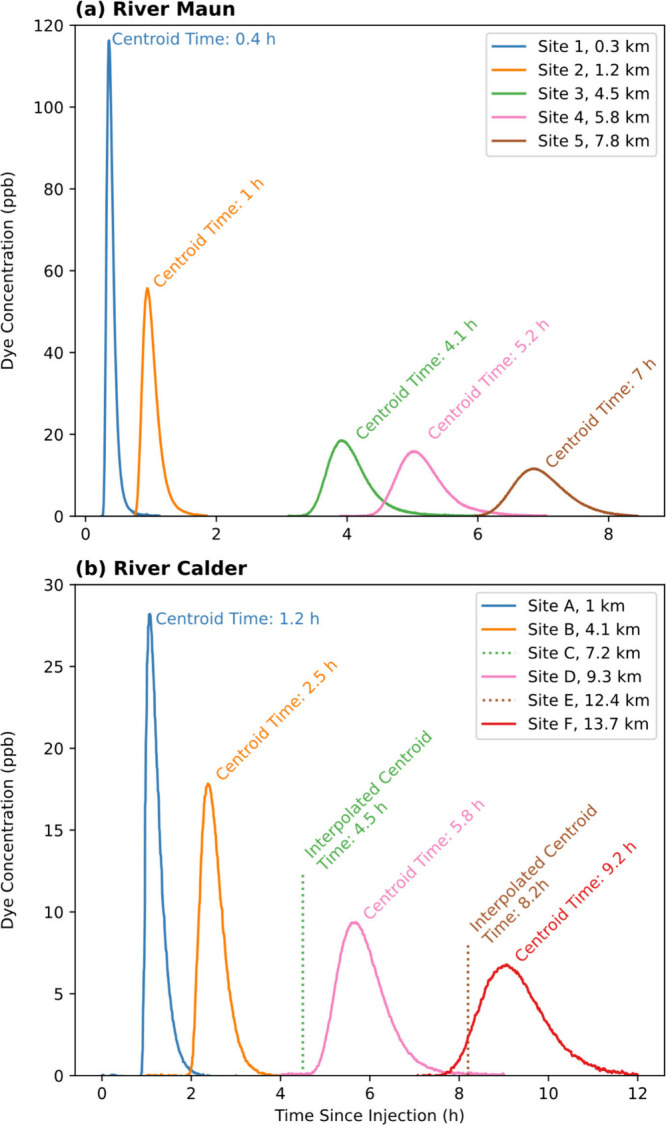
Rhodamine WT concentrations against time since injection at sampling sites downstream of (a) Mansfield STP in the River Maun and (b) Dewsbury STP in the River Calder. Dashed lines indicate sampling sites where the centroid time was linearly interpolated from adjacent stations. Distances are from the STP outfall locations.

### Sucralose

Immediately downstream of Mansfield STP (Site 1), the concentration of sucralose in the River Maun was 51 μg L^–1^, which decreased to 37 μg L^–1^ at Site 5 (Figure 3). The observed concentrations of sucralose closely match (*R* ^2^ = 0.95) predicted concentrations based on dilution with discharge obtained through dilution gauging, assuming no degradation (*k* = 0) over the study period (eq 4). This confirms the assumption that sucralose is not significantly degraded or lost from the river water and strongly supports its use as a persistent tracer to account for dilution effects (eq 5).

**Figure 3:**
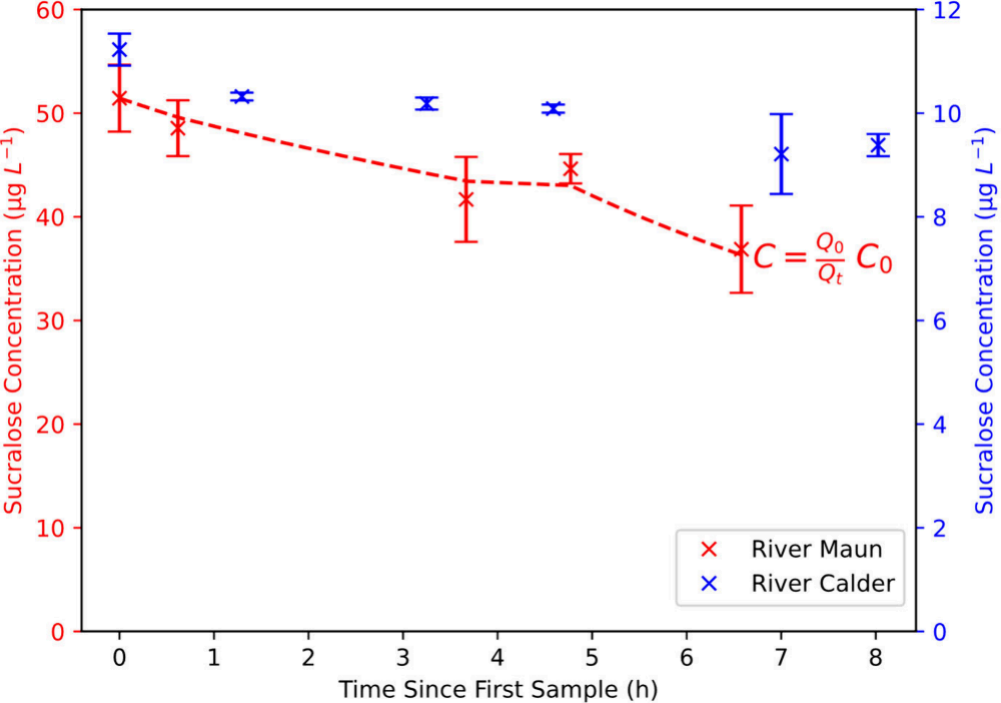
Sucralose concentrations in the River Maun (red) and River Calder (blue) against the solute travel time. The dashed line indicates predicted concentrations of sucralose in the River Maun based on dilution with measured flow data obtained through dilution gauging, assuming no degradation (*k* = 0) over the study period. An equivalent line is not shown for the River Calder due to hydrological uncertainties in this river (see the text).

In the River Calder, sucralose concentrations decreased slightly from 11 μg L^–1^ immediately downstream of Dewsbury STP (Site A) to 9.4 μg L^–1^ at Site F, suggesting a 17% increase in *Q* (Figure 3). Agreement between dilution estimates based on sucralose concentrations (*C*_*t*,PER_/*C*_0,PER_) and those based on dilution gauging (*Q*_0_/*Q*_*t*_) were poorer for the Calder than for the Maun (*R* ^2^ = 0.79). This may be the result of apparent abstractions in the Calder. In both catchments, flow accretion would be expected due to baseflow contributions (groundwater discharge) and hillslope runoff. However, in the Calder estimates of *Q* from dilution gauging decreased by ∼5% between Site A (*Q* = 25.3 m^3^ s^–1^) and Site F (*Q* = 23.9 m^3^ s^–1^). Although, this apparent reduction in *Q* is within the error typically reported for dilution gauging (approximately 10% ^63,64^ ), the fact that an increase in *Q* was not detected suggests that some water may have been abstracted from the river along the study reach to augment the Calder and Hebble canal, which is replaced by approximately equivalent baseflow contributions with distance downstream. With the exception of NH_4_^+^, baseflow and hillslope contributions to discharge are unlikely contain wastewater contaminants. Contributions from decentralized wastewater treatment systems, such as septic tanks, are believed to be minimal because the vast majority (∼96%) of the population of England and Wales is served by centralized wastewater collection and treatment.^65^ This complexity supports the use of chemical benchmarking with sucralose (eq 5) as the most appropriate approach for adjusting values of *k* for NH_4_^+^, caffeine, and LAS for dilution.

### NH_4_^+^, Caffeine, and LAS

In all samples, concentrations of sucralose, NH_4_^+^, caffeine, and LAS were significantly greater than their respective LOQs. The in-stream removal of NH_4_^+^, caffeine, and LAS differed substantially between the two rivers (Figure 4). Concentrations of NH_4_^+^ in the River Maun decreased from 0.48 mg N L^–1^ at Site 1 to 0.16 mg N L^–1^ at Site 5. This results in a fitted first-order nitrification rate constant (*k*) of 0.177 h^–1^ (employing chemical benchmarking to account for dilution), corresponding to a first-order half-life (*t*_1/2_) of 3.9 h. Model accuracy was high, with an RMSE value of 4.27 × 10^–2^ mg N L^–1^. This half-life is consistent with nitrification rate constants in other shallow streams. For example, a half-life of 2.4 h was reported for the Red Beck, a small tributary stream in the Calder catchment.^37^ Similarly, a value of 2.97 h was reported by McAvoy et al.^44^ for nitrification in a shallow river in the Philippines.

**Figure 4:**
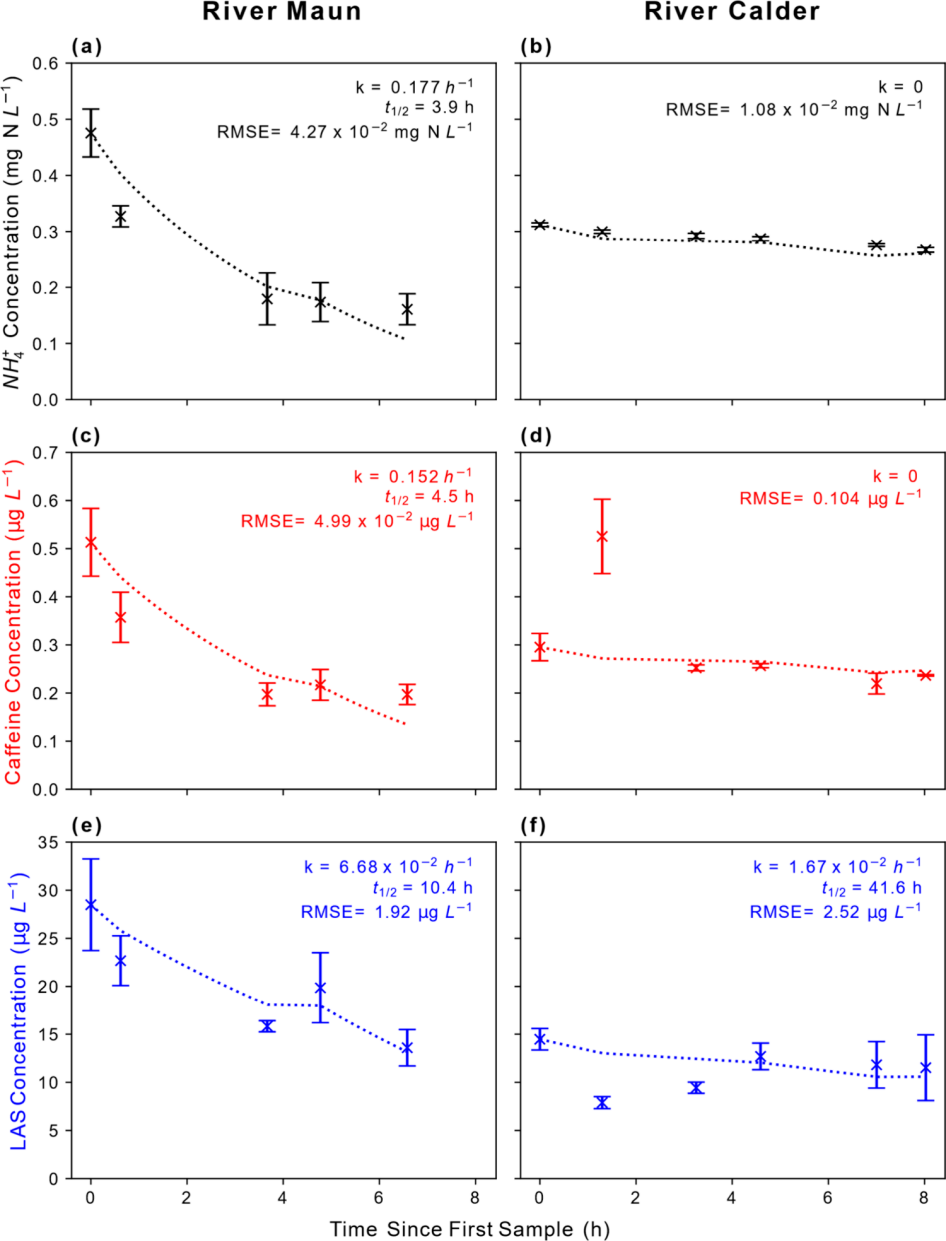
Concentrations of ammonium (a and b), caffeine (c and d), and linear alkylbenzenesulfonate (e and f) in the River Maun (left panels) and River Calder (right panels) against the solute travel time. Dashed lines show dilution-corrected first-order models (eq 5). The fitted rate constants (*k*), first-order half-lives (*t*_1/2_), and RMSE of fitted first-order transformation models are also shown. Error bars represent standard errors.

In contrast, NH_4_^+^ concentrations in the River Calder decreased only slightly from 0.31 mg N L^–1^ at Site A to 0.27 mg N L^–1^ at Site F. When accounting for dilution, these data imply no net loss of NH_4_^+^ over the monitored reach in this period (i.e., a fitted value for *k* of 0 and a model RMSE value of 1.08 × 10^–2^ mg N L^–1^). The difference in nitrification rates was partially explained by differences in the river water temperature during sampling (Table 2). In order to enable a fair comparison between nitrification rates, *k* values in the River Maun were normalized to a reference temperature, *T*_r_, of 9.5 °C (the average water temperature in the River Calder). The temperature-corrected value of *k* for NH_4_^+^ oxidation in the Maun was 3.19 × 10^–2^ h^–1^ (*t*_1/2_ = 21.7 h).

**Table 2:** *In Situ* Water Quality Parameters Measured in the Rivers Maun and Calder (Mean for All Sites ± Standard Error)

river	*Q* (m^3^ s^–1^)	temp (°C)	pH	DO (mg L^–1^)	DO (%)	EC (μS cm^–1^)
Maun	0.94 ± 0.1	16.7 ± 0.29	7.96 ± 0.02	8.44 ± 0.12	89.8 ± 1.1	686 ± 35
Calder	24.2 ± 0.3	9.5 ± 0.06	8.63 ± 0.04	11.0 ± 0.05	96.6 ± 0.4	288 ± 2.8

Caffeine concentrations in the River Maun decreased from 0.51 μg L^–1^ at Site 1 to 0.20 μg L^–1^ at Site 5. The fitted biodegradation rate constant (*k*) was 0.152 h^–1^ (*t*_1/2_ = 4.5 h). When normalized to *T*_r_ (9.5 °C), the adjusted value for *k* was 7.61 × 10^–2^ h^–1^ (*t*_1/2_ = 9.1 h). In contrast, caffeine concentrations in the River Calder decreased from 0.30 μg L^–1^ at Site A to 0.24 μg L^–1^ at Site F. This decrease was not sufficient to imply any biodegradation, after allowing for dilution (fitted value for *k* = 0). Again, the model performance for both rivers was good, with RMSE values of 4.99 × 10^–2^ and 0.104 μg L^–1^ in the Rivers Maun and Calder, respectively. The slightly higher RMSE in the River Calder was caused by an unexplained spike in the caffeine concentration at Site B (0.53 μg L^–1^). Note that there were no known sources of domestic wastewater between Sites A and B, and combined sewer overflows were unlikely to have been in operation (dry weather). It is possible that this may have been the result of an analytical artifact. To the best of our knowledge, in-stream removal rates for caffeine have not been previously measured elsewhere. However, rapid biodegradation of caffeine is expected. Bradley et al.^66^ reported half-lives for caffeine between 5.3 and 24 h in a laboratory microcosm experiment (usually longer than those observed in the field^10,35^ ). This arises from a combination of factors including the acclimation and increased complexity of natural biofilms, which generally enhances their ability to degrade perennially present contaminants (e.g., from steady-state emission of wastewater).^67,68^

LAS concentrations in the River Maun decreased from 28 μg L^–1^ at Site 1 to 14 μg L^–1^ at Site 5. The fitted biodegradation rate constant (*k*) was 6.68 × 10^–2^ h^–1^ (*t*_1/2_ = 10.4 h), which is equivalent to 3.35 × 10^–2^ h^–1^ (*t*_1/2_ = 20.7 h) at *T*_r_ (9.5 °C). In the River Calder, LAS concentrations decreased from 14 μg L^–1^ at Site A to 12 μg L^–1^ at Site F. Despite the minor decline in concentration, the first-order fit (eq 5) was significant (*p* < 0.05) with a derived rate constant of 1.67 × 10^–2^ h^–1^ (*t*_1/2_ = 42 h). The model performance was acceptable, with RMSE values of 1.92 μg L^–1^ in the River Maun and 2.52 μg L^–1^ in the River Calder. The slightly higher RMSE in the Calder was largely caused by high residuals at Sites B and C resulting from lower LAS concentrations compared with the overall trend. This may have been influenced by the discharge of treated wastewater from Horbury STP (Figure 1), which could have slightly elevated riverine concentrations between stations C and D. This was explored using a simple mixing calculation in which the load at Horbury was estimated from the population served (16000), assuming a domestic water use of 150 L cap^–1^ day^–1^, a mean LAS use of 3.18 g cap^–1^ day^–1^, and a 99% removal rate of LAS during the wastewater treatment process (see the Supporting Information).^69,70^ This calculation suggested that the increase in the LAS concentration downstream of Horbury would be in the region of 0.2 μg L^–1^. This minor influence is consistent with data for sucralose (Figure 3), NH_4_^+^, and caffeine (Figure 4), which do not indicate a significant wastewater input from Horbury. The first-order half-lives reported for LAS in both rivers (10.4–41.6 h) were similar in magnitude to those reported in other rivers. For example, half-lives between 0.9 and 36 h have been reported for rivers in the U.K.,^37^ USA,^71,72^ Italy,^73,74^ Japan,^11^ Laos^36^ and the Philippines.^44^ It should be noted that these rate constants are biased toward easily accessible (wadable) streams, which we anticipate to be lower than those for larger rivers.

### Discussion

Microbially mediated transformation rates (nitrification and biodegradation) were consistently faster in the River Maun, which is a small and shallow stream, compared to the River Calder. The ratio of normalized LAS half-lives between the Maun (20.7 h) and Calder (41.6 h) was 0.49, closely aligning with the range of estimated depth ratios (0.23–0.42). This supports the proposed hypothesis that microbial transformation rate coefficients are inversely proportional to *R*. Note that the half-life ratios of NH_4_^+^ and caffeine could not be calculated since *k* was not significantly different from zero in the River Calder. However, this also supports the proposed hypothesis qualitatively. Of course, the difference in observed rates may not be entirely attributable to differences in *R*. However, (1) we accounted for the effects of temperature, and (2) measured DO concentrations (Table 2) were above the thresholds that limit aerobic nitrification and biodegradation (4 mg L^–1^ at most).^75–78^ The small difference in pH (Table 2) was also unlikely to have affected the microbial function.^79–81^ However, there are likely to have been differences in biofilm community composition and function in each river, which may have affected microbial transformation rates.^24,68,82^ Unfortunately, it was beyond the scope of this work to characterize the biofilm in each river, but further work should be directed to assessing biofilm community composition.

While we did not observe significant nitrification or caffeine biodegradation in the River Calder, these processes will certainly have been operating in this system and may have been measurable over a longer travel time. This would have required sampling over a greater distance, which is often challenging due to increased likelihood of additional tributary and wastewater inputs along the river. For example, in the Calder, monitoring was curtailed upstream of Wakefield STP, the next major STP on the system (Figure 1). That said, sucralose was shown to be a good marker for domestic wastewater contribution and downstream dilution, which suggests that benchmarking could be used to disentangle the complexities of pollutant degradation tracking over larger spatial scales.

It is important to note that the reported values for *k* represent removal from all potential loss mechanisms, including microbially mediated transformation, volatilization, and sorption to sediment. For caffeine and LAS, volatilization is likely to be negligible due to their very low Henry’s law constants (3.63 × 10^–6^ and 6.35 × 10^–3^ Pa m^3^ mol^–1^, respectively). For NH_4_^+^, volatilization of free (un-ionized) ammonia (which coexists with NH_4_^+^ as part of total ammoniacal nitrogen) is possible. However, we calculated that only 3% and 7% of total ammoniacal nitrogen would have been in the form of free ammonia in the Maun and Calder, respectively (see the Supporting Information). Similarly, some water column losses of NH_4_^+^, caffeine, and LAS due to sorption are possible. However, sorption is generally not considered a significant net removal mechanism for wastewater pollutants in most rivers and streams because the discharge of these pollutants is approximately continuous, allowing for the establishment of thermodynamic equilibrium partitioning between the aqueous phase and suspended and bed sediment.^6,83–86^

For NH_4_^+^, removal could also be influenced by plant uptake and the conversion of mineral nitrogen to organic nitrogen (immobilization).^85,87,88^ However, rates of plant uptake are predicted to be low in lotic systems, and there was limited in-stream vegetation in each river.^89,90^ Moreover, we assume that rates of immobilization were less than or equal to rates of mineralization (the conversion of organic nitrogen to mineral nitrogen) resulting in no net immobilization. This will depend, at least in part, on the C:N ratio of organic matter in the water column and in the sediment substrate.^91,92^ It should also be acknowledged that diffuse sources of NH_4_^+^, unrelated to wastewater, such as agricultural runoff, could have entered both rivers, potentially affecting the reported values for *k*.^93,94^

DO concentrations in both rivers were always relatively high (>7.97 mg L^–1^ in the Maun and >10.91 mg L^–1^ in the Calder, corresponding to high levels of saturation: >86% in the Maun and >95% in the Calder). These levels are unlikely to inhibit either nitrification (DO thresholds for nitrification inhibition as high as 4 mg L^–1^ have been reported but are more typically <2 mg L^–1^)^75^ or biodegradation (inhibition thresholds for biodegradation are typically <1–2 mg L^–1^)^76–78^ in the water column or at the sediment–water interface. However, DO concentrations are usually depressed in river sediments,^95^ and this could influence overall system behavior if hyporheic exchange is important.^96^

Our assumption of temperature-adjusted first-order kinetics to describe in-stream contaminant transformations is commonly adopted in both operational river water quality models, such as SIMCAT,^97^ iSTREEM,^98^ and GREAT-ER,^99^ and in field and laboratory studies of biodegradation.^5,7^ However, for some contaminants, concentration-independent rates (zero-order kinetics) may be observed at high concentrations, necessitating the use of saturation (Michaelis–Menten) kinetics. This does not appear to be required in our case. Similarly, describing kinetic dependence on microbial growth is not appropriate in our systems because microbial populations in riverine biofilms can be assumed to be in steady state.

### Environmental Implications

In our study, each monitored river reach is considered to be a relatively homogeneous system with a similar hydraulic geometry along the whole reach. We expect the effects of hydraulic radius between stations in each reach to be relatively minor at the scales investigated here, which allows a single rate constant to be derived for each river. Rather than explaining small differences between stations, we argue here that the effects of the channel geometry are manifested at the macro (whole-system) level, explaining the differences between highly contrasting reaches in different river systems (in this case the Maun and Calder) or between different stages in the whole river long profile at the catchment scale. Such differences will be most apparent at the large catchment scale. Our data suggest that the channel geometry significantly affects the in-stream transformation rate constants and, by extension, environmental exposure profiles for contaminants and pollutant exports from fluvial to marine systems. Channel geometry characteristics could be incorporated into models, predicting pollutant behavior in rivers quite easily. For example, the nitrification and biodegradation rate constants (*k*) could be modified by depth, such that

7 $$k=\frac{k_{\mathrm{ref}}}{d}$$

where *k*_ref_ is a reference value for *k* (which may be derived from measured rates obtained in flume or laboratory experiments or from dye tracing studies, such as those reported here). The implications of this modification to *k* can be illustrated via a simple first-order model describing chemical removal (*r*, %) downstream of a point source:

8 $$r=1-\exp\left(-\frac{k_{\mathrm{ref}}}{d}t\right)$$

For simplicity, this model assumes no dilution or change in depth with distance downstream. We used this model to predict the removal of NH_4_^+^, caffeine, and LAS in hypothetical rivers with different channel depths. *k*_ref_ values were calculated from rate constants measured in the River Maun (normalized to *T*_r_) and the UK CEH bankfull estimate of *d* (1.25 m; Table 1). Illustrative results showing chemical removal over 24 h for four different depth profiles ranging from 0.5 to 5 m are presented in Figure 5. This range of depths was based on the range of UK CEH bankfull *d* estimates.^39^

**Figure 5:**
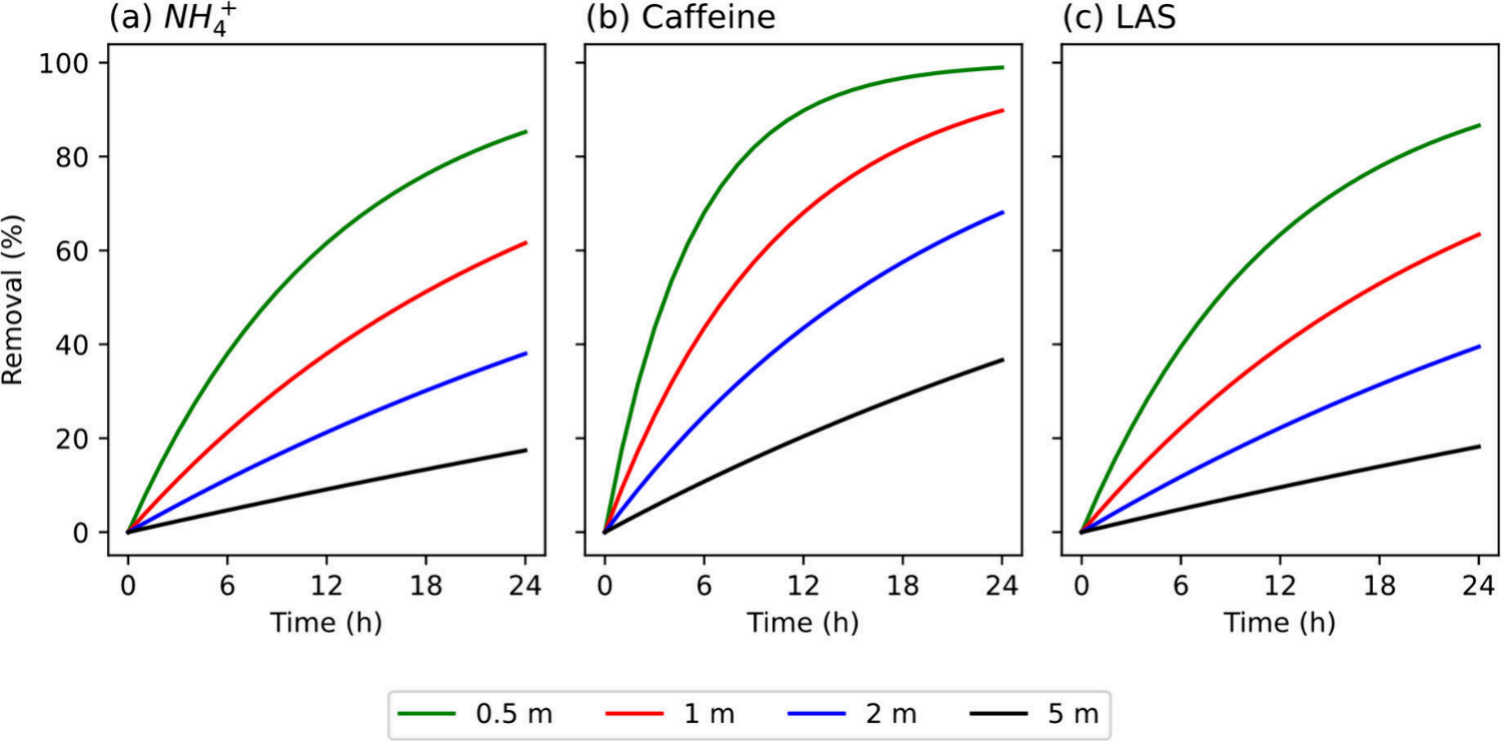
Predicted removal of (a) NH_4_^+^, (b) caffeine, and (c) LAS at different channel depths (0.5, 1, 2, and 5 m).

There was a substantial difference in the predicted removal of NH_4_^+^, caffeine, and LAS with different assumed channel depths. The largest difference in chemical removal occurred when *k*_ref_ was high. For example, *k*_ref_ for caffeine was 9.51 × 10^–2^ h^–1^, which resulted in a predicted removal of 99% over 24 h in the 0.5 m channel and 37% in the 5 m channel. In comparison, *k*_ref_ for NH_4_^+^ was 3.98 × 10^–2^ h^–1^, which results in predicted removals of 85% in the 0.5 m channel and 17% in the 5 m channel. These calculations support our conclusions from field-based observations in the Rivers Maun and Calder that major reductions in chemical loss rates are likely as the scale of channel dimensions increases. These differences will be most pronounced in very large river basins with long residence times and large systematic increases in channel dimensions from the headwaters to the tidal limit.

### Conclusions

Our data demonstrate clear differences in microbially mediated wastewater pollutant transformation rates in two rivers with contrasting morphologies. These differences support the hypothesis that transformation rate constants for many pollutants will be inversely proportional to *R*. Although evidence for such geomorphological controls exists for nitrification and denitrification, there has been, hitherto, a paucity in field data comparing the biodegradation rate constants for organic contaminants in rivers. Similar hydraulic geometry controls can be expected for other removal mechanisms such as photodegradation (which will decrease with depth due to the extinction of light) and volatilization (which occurs only across the air–water interface, implying that the overall rate constant will also decrease as the water depth increases). These findings highlight the need to consider river channel geomorphology in higher-tier chemical exposure models and associated risk assessments. Most in-stream exposure models employ a single rate constant (*k*) for different loss mechanisms across all reaches in a channel network. This assumption is unlikely to be appropriate at the large catchment scale because *k* will be modified by systematic and major changes to the channel shape and size. Further research on other rivers in different seasons is needed to refine this understanding. Many rivers have suitable reaches in which this type of tracing exercise could be conducted in order to supplement the data reported here and reinforce the generality of our findings. In addition, such research would benefit from characterizing the structure and functional capabilities of biofilm communities, which is beyond the scope of the work presented here.

## Supplementary Material





## Notes

Justification that the hydraulic radius is approximately equal to depth, sampling point coordinates, equations used to calculate the tracer centroid and river discharge, river stage at Site B from the Environment Agency, LC-MS/MS conditions for the analyses of caffeine, LAS, and sucralose (e.g., column type, mobile phase, and MS source conditions), LC-MS/MS method development and validation, estimated emissions of LAS from Horbury STP, and equations used to calculate the fraction of free (un-ionized) ammonia (NH_3_) in water (PDF)
Raw data and source code used to produce the figures (ZIP)

## †

Yorkshire Water Services, Bradford BD6 2SZ, U.K. (robert.newbould@yorkshirewater.co.uk)
