# Supplementary material for: Channel Geometry Controls on Chemical Behavior in Rivers: Insights from a Comparative Field Study
Source: ACS ES T Water. 2025 Sep 9;5(10):5785–96. doi: 10.1021/acsestwater.4c01203 (PMC12519465; doi:10.1021/acsestwater.4c01203)
Supplement: Supplementary file 1 [file ew4c01203_si_001.pdf]

Supporting Information For

Channel Geometry Controls on Chemical Behavior in Rivers: Insights from a Comparative Field Study

Robert A. Newbould[1*#], D. Mark Powell[1], Juliet Hodges[2], Alexandre Teixeira[2], Ian Guymer[3], Michael J. Whelan[1]

[1] School of Geography, Geology and the Environment, University of Leicester, Leicester, LE1 7RH, UK

[2] Safety, Environmental and Regulatory Science, Unilever, Colworth Science Park, Sharnbrook, MK44 1LQ, UK

[3] School of Mechanical, Aerospace and Civil Engineering, The University of Sheffield, Sheffield, S1 3JD, UK

[*] Corresponding Author: ran14@le.ac.uk

[#] Present Address: Yorkshire Water Services, Bradford, BD6 2SZ, UK (robert.newbould@yorkshirewater.co.uk)

Supplementary Information: Comparison of Hydraulic Radius and Depth

In natural channels, hydraulic radius (*R:* the ratio of channel cross-sectional area to wetted perimeter) is approximately equal to channel depth (*d*)[1,2]. In this paper, we used three approaches to characterise *R*, all of which assumed that *R* is approximated by *d*. To justify this assumption, here we present two approaches to estimate *R*, both of which assume a rectangular channel:

$$R = \frac{w.d}{w+2d} \quad \text{(S1)}$$

where *w* is channel width (m). The first approach to estimate *R* using Equation S1 used the simple relationship:

$$Q = w.d.v \quad \text{(S2)}$$

where v is velocity (m s^{-1})[3]. Q and v were obtained through dilution gauging and solute travel time, respectively, while w was measured using satellite imagery. This approach is consistent with that used in the main text to estimate *d*.

The second approach to estimate *R* utilised gridded estimates of bankfull *w* and *d* from the UK Centre for Ecology and Hydrology[4]. This data product was derived from a hydraulic geometry relationship between *w* or *d* and the product of catchment area (A, km^2) and mean annual rainfall (R_f, mm), which is a proxy for Q:

$$w = 0.0042\, A^{0.409}\, {R_f}^{0.86} \quad \text{(S3)}$$

$$d = 0.02643\, A^{0.202}\, {R_f}^{0.482} \quad \text{(S4)}$$

These relationships were calibrated against historical survey data, with full details available in Davies et al.[4]. Again, this approach is consistent with that used in the main text. The estimates of *R* using the approaches described above were very similar to the estimates of *d* described in the main text (Table S1). This supports the assumption that *R* is approximately equal to *d*.

Table S1 – Estimates of *R* and *d* in the River Maun and River Calder

	Maun		Calder	
**Approach**	***R* (m)**	***d* (m)**	***R* (m)**	***d* (m)**
Hydraulic Geometry	0.31	0.34	1.34	1.45
UK CEH	0.83	1.25	2.45	3

Supplementary Information: Coordinates of Sampling Sites

Table S2 – Sampling point coordinates in the River Calder (Sites 1-5) and River Maun (Sites A-F) along with distance downstream from respective effluent discharge points

Site	Coordinates	Distance Downstream (km)
1	53°09'28.4"N, 1°10'56.5"W	0.3
2	53°09'46.9"N, 1°10'26.9"W	1.2
3	53°10'26.0"N, 1°07'51.6"W	4.5
4	53°10'45.5"N, 1°06'57.7"W	5.8
5	53°11'02.8"N, 1°05'31.4"W	7.8
A	53°40'06.7"N, 1°35'34.5"W	1
B	53°39'19.2"N, 1°33'45.2"W	4.1
C	53°39'10.1"N, 1°31'57.6"W	7.2
D	53°39'48.0"N, 1°30'30.3"W	9.3
E	53°40'34.7"N, 1°29'31.6"W	12.4
F	53°40'30.8"N, 1°28'25.7"W	13.7

Supplementary Information: Calculating Tracer Centroid and River Discharge

In order to calculate the rhodamine tracer centroid (centre of mass) and to estimate river discharge through dilution gauging, the following equations were used:

$$t_c = \frac{\sum_{i=1}^{n}(C_i.\Delta t).t_i}{\sum_{i=1}^{n} C_i.\Delta t} \qquad \text{(S1)}$$

$$Q = M\,(\sum_{i=1}^{n} C_i.\,\Delta t) \qquad \text{(S2)}$$

where t_c is the tracer centroid time (s), C_i is the dye concentration at the i-th time interval (μg L^{-1}), Δt is the duration of each time interval (s), t_i is the time since injection at the i-th time interval (s), n is the total number of time intervals, Q is river discharge (m^3 s^{-1}) and M is the mass of dye injected (mg) (see Figure S1)[5,6].

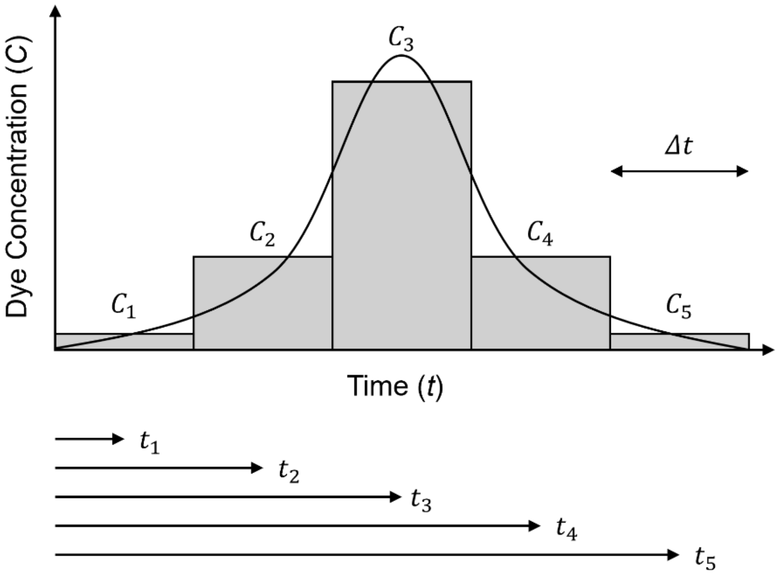

Figure S1 – Illustration of tracer concentration, C_i, over time, t_i

Supplementary Information: River Calder Stage Data

In the River Calder, dye tracing was conducted overnight on 19 February 2024 and water samples were collected the following day, 20 February 2024, using travel times determined from the dye trace. This approach is acceptable under steady flow conditions, which was confirmed with stage data at Site B from the Environment Agency (environment.data.gov.uk/hydrology; Figure S2). River stage represents the height of a river relative to a fixed point on or near the riverbed (local datum).

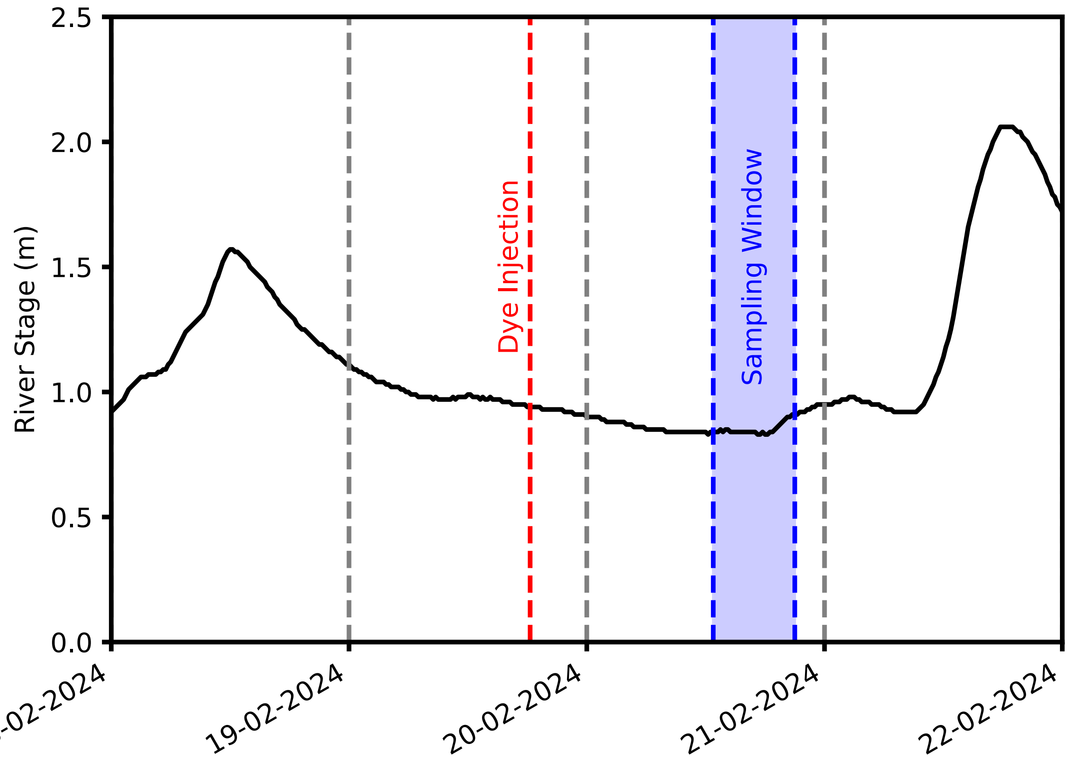

Figure S2 – River stage (black) before, during and after dye injection (red) and water sampling (blue)

Supplementary Information: LC-MS/MS Conditions for Analyses of Caffeine

Instrument: Agilent 1290 series LC-MS/MS System with 6495 triple quadrupole mass spectrometer

Clean Up Column: Thermo Hypercarb 3umm 2.1 x 10 mm guard column (placed in between pump outlet and injector flow)

Analytical Column: XBridge BEH shield RP18 2.5 µm, 2.1 x 50 mm column XP + equivalent 5 mm guard-column

Column Temperature: 30°C

Injection Volume: 10 µL

Flow Rate: 0.7mL/min

Mobile Phase: Water (95%)/MeoH (5%) + 0.1% Formic acid / Methanol (95%)/Water (5%) + 0.1% Formic acid (B)

Gradient:

**min**	0	0.5	1.0	1.2	2.0	5.0	5.5	7.0
**%A**	100	100	50	20	0	0	100	100

MS Source Conditions:

- Source Gas Temperature: 230°C
- Sheath Gas Temperature: 400°C
- Capillary Voltage: 2500v
- Nozzle Voltage: 500v
- High Pressure RF: 130v
- Low Pressure RF: 120v

MS/MS Conditions:

Compound	Precursor Ion (m/z)	Product Ion (m/z)	Collision Energy (eV)
Caffeine	195.1	138.1	10
Caffeine $^{13}C_3$ *	198.1	140.1	
Diclofenac	296.0	214.0	
Diclofenac $^{13}C_6$*	302.0	221.0	

* internal standard

Limit of Quantification: 0.1 µg L^{-1} for caffeine and 0.5 µg L^{-1} for diclofenac

Supplementary Information: LC-MS/MS Conditions for Analyses of Sucralose and LAS

Instrument: Agilent 1290 series LC-MS/MS System with 6495 triple quadrupole mass spectrometer

Clean Up Column: Thermo Hypercarb 3umm 2.1 x 10 mm guard column (placed in between pump outlet and injector flow)

Analytical Column: XBridge BEH shield RP18 2.5 µm, 2.1 x 50 mm column XP + equivalent 5 mm guard-column

Column Temperature: 30°C

Injection Volume: 10 µL

Flow Rate: 0.7mL/min

Mobile Phase: Water (95%)/MeoH (5%) + 5mM Ammonium Formate/Methanol (95%)/Water (5%) + 5 mM Ammonium Formate (B)

Gradient:

**min**	0	0.5	1.5	3.0	5.0	5.1	7.0
**%A**	100	100	30	0	0	100	100

MS source conditions:

- Source Gas Temperature: 230°C
- Sheath Gas Temperature: 400°C
- Capillary Voltage: 3000v
- Nozzle Voltage: 0v
- High Pressure RF: 90v
- Low Pressure RF: 60v

MS/MS conditions:

Compound	Precursor Ion (m/z)	Product Ion (m/z)	Collision Energy (eV)
Sucralose*	441	395	6
C_{10} LAS	297.2	183	38
C_{11} LAS	311.2	183	38
C_{12} LAS	325.2	183	38
C_{12} AS d25 **	290.5	98.1	38

* formate ion $[M+CHO_2]^-$ to molecular ion $[M-H]^-$ transition; ** internal standard

Limit of Quantification:

Sucralose	C_{10} LAS	C_{11} LAS	C_{12} LAS
0.5 µg L^{-1}	0.9 µg L^{-1}	2.2 µg L^{-1}	1.6 µg L^{-1}

Supplementary Information: LC-MS/MS Method Development and Validation

The extracted ion chromatograms for each compound and internal standard were obtained using the LC-MS/MS conditions stated above with 100 µg L^{-1} calibration standards (Figure S3). Water samples were also analysed for diclofenac, as shown by Figure S3, but measured concentrations were mostly below the limit of quantification (LOQ). Therefore, diclofenac was excluded from our analysis. Diclofenac (≥ 98.5%) and its internal standard, diclofenac-acetophenyl ring-$^{13}C_6$ (≥ 99%), were obtained from Sigma-Aldrich (Gillingham, UK).

(a) Positive Ion Mode

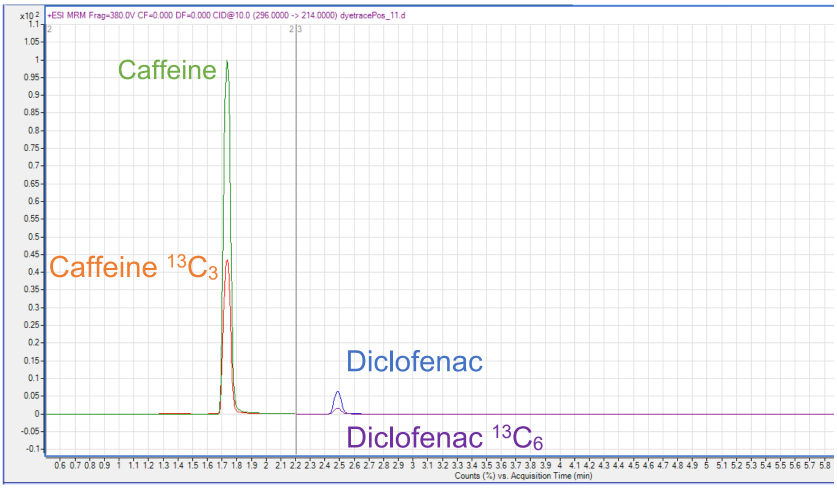

(b) Negative Ion Mode

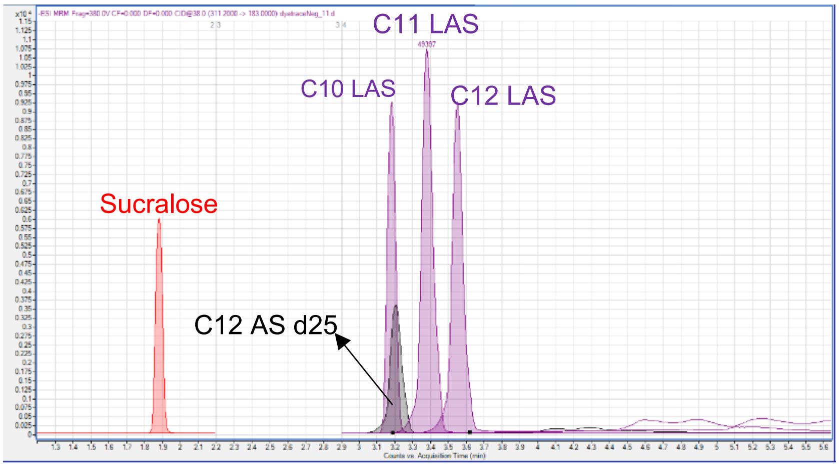

Figure S3 – Overlay of extracted ion chromatograms for (a) positive ion mode for the analysis of caffeine and its internal standard, and (b) negative ion mode for the analyses of sucralose, C_{10}-C_{12} LAS and the internal standard (C_{12} AS d25)

Calibration curves were generated for each compound (Figure S4). Each calibration standard was run in duplicate, with a 1/x fit. Quantification was performed with internal standard calibration. An analytical run was considered valued if R^2 > 0.99 and the back calculated concentration of each standard was within 80 to 120% of the nominal value. Quality control standards, prepared separately at two different concentrations covering the linear range, were injected throughout each run. The acceptance criteria were a coefficient of variation (CV) of less than 20% and a calculated concentration within 20% of the nominal value. For caffeine and sucralose, the LOQ was determined as the lowest concentrated standard that produced a signal-to-noise ratio > 10 and a CV < 25% (n = 3). Due to the widespread use of LAS, high background levels were observed. The LOQ for each LAS analogue was determined as the lowest concentration that was consistently twice the average response in ultrapure water.

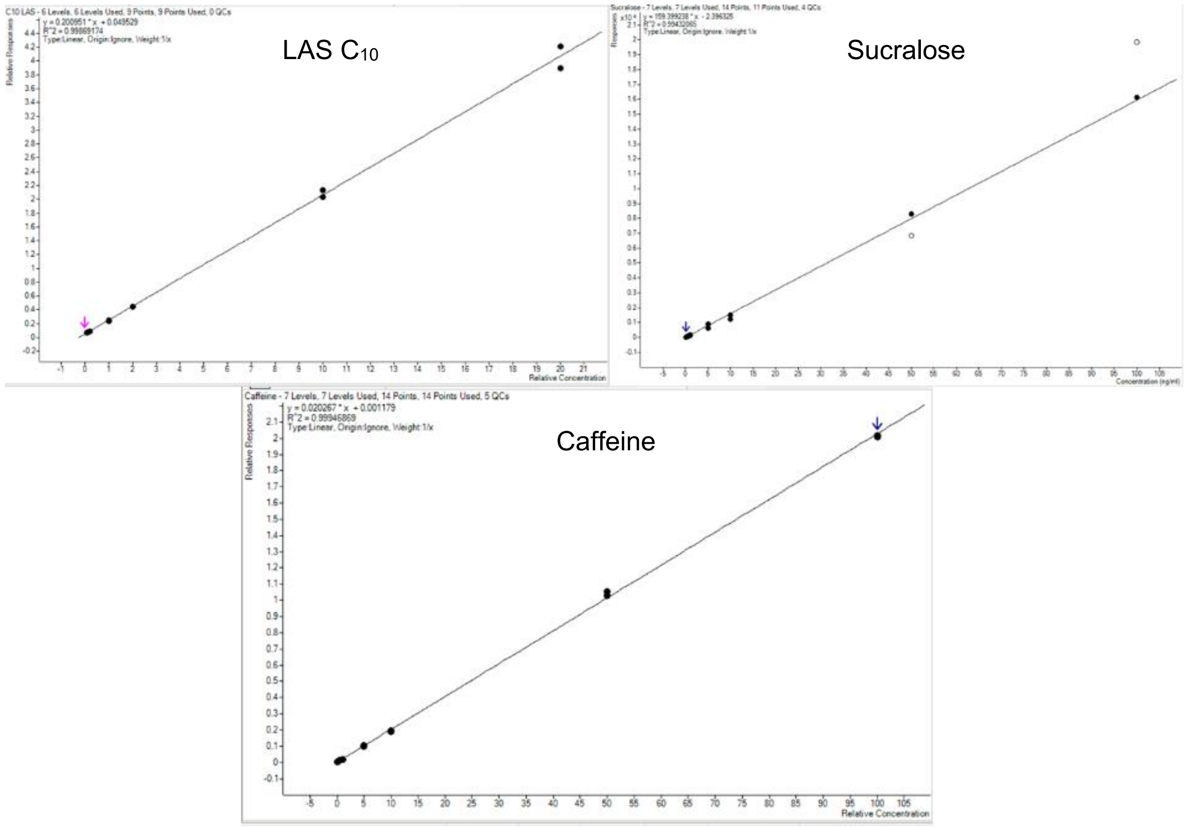

Figure S4 – Calibration curves for C_{10} LAS, sucralose and caffeine

Supplementary Information: Estimated Emissions of LAS from Horbury STP

To assess the potential contribution of Horbury STP to elevated LAS concentrations downstream of Site B in the River Calder, a simple mass balance and mixing model was considered:

$$C_{DS} = \frac{Q_{US}\,C_{US} + Q_{eff}\,C_{eff}}{Q_{US} + Q_{eff}} \qquad \text{(S3)}$$

where C is LAS concentration (µg L^{-1}), Q is discharge (m^3 s^{-1}) and the subscripts US, DS and eff represents the river upstream and downstream of Horbury STP and of the effluent itself[7]. Q_{eff} and C_{eff} were estimated based on the population served (16,000), assuming a domestic water use of 150 L cap^{-1} day^{-1}, a mean LAS use of 3.18 g cap^{-1} day^{-1} and 99% removal of LAS during the wastewater treatment process[8]. The estimated concentration of LAS downstream of Horbury STP (C_{DS}) was 8.1 µg L^{-1}, only 0.2 µg L^{-1} higher than the LAS concentration at Site B (7.9 µg L^{-1}). This suggests that the contribution of Horbury STP to LAS concentrations downstream of Site B was small. This is consistent with the sucralose, ammonium and caffeine data. However, it is important to note that there is some uncertainty around C_{DS} as wastewater effluent discharge exhibits significant temporal and diurnal variability[9,10], which Equation S3 does not account for. The estimated dilution factor (DF) of Horbury STP based on Equation S3 was 903. This is the same order of magnitude as the DF of 504 estimated by HydroWASTE[11] at mean flow. The higher DF was expected as the river discharge during sampling exceeded mean flow.

Supplementary Information: Calculating the Fraction of Free Ammonia (NH_3)

In order to calculate the fraction of free ammonia (NH_3) in the Maun and Calder, the following equations were used:

$$f_{NH_3/NH_4^+} = \frac{1}{1+10^{(\mathrm{pKa}-\mathrm{pH})}} \qquad \text{(S4)}$$

$$\mathrm{pKa} = 0.09018 + 2729.92/T \qquad \text{(S5)}$$

where T is temperature in K[12,13]. The mean temperatures in the Maun and Calder were 16.7°C (289.85 K) and 9.5°C (282.65 K), respectively, with average pH values of 7.96 and 8.63. It was calculated that 3% and 7% of the total ammoniacal nitrogen was estimates to have been in the form of NH_3, respectively.

References

(1) Leopold, L. B.; Maddock, T. *USGS Professional Paper 252: The Hydraulic Geometry of Stream Channels and Some Physiographic Implications*; US Government Printing Office: Washington, 1953. https://doi.org/10.3133/pp252.

(2) Richards, K. *Rivers: Form and Process in Alluvial Channels*; Routledge: London, 1982. https://doi.org/10.4324/9781003465799.

(3) Ferguson, R. I. Hydraulics and Hydraulic Geometry. *Prog. Phys. Geogr. Earth Environ.* **1986**, *10* (1), 1–31. https://doi.org/10.1177/030913338601000101.

(4) Davies, H. N.; Rameshwaran, P.; Bell, V. A.; Dadson, S. Spatially Consistent Physical Characteristics of UK Rivers: 1-Km Data. *Geosci. Data J.* **2024**, *11*, 284–291. https://doi.org/10.1002/gdj3.209.

(5) Kilpatrick, F. A.; Cobb, E. D. *Measurement of Discharge Using Tracers*; US Government Printing Office: Washington, 1985.

(6) Hubbard, E.; Kilpatrick, F.; Martens, L.; Wilson, J. *Measurement of Time of Travel and Dispersion in Streams by Dye Tracing*; US Government Printing Office: Washington, 1982.

(7) Warn, A. E.; Brew, J. S. Mass Balance. *Water Res.* **1980**, *14* (10), 1427–1434. https://doi.org/10.1016/0043-1354(80)90007-X.

(8) HERA. *Linear Alkylbenzene Sulphonate*; 2013. https://www.heraproject.com/files/HERA-LAS%20revised%20April%202013%20Final1.pdf (accessed 2024-06-26).

(9) Whelan, M. J.; Gandolfi, C.; Bischetti, G. B. A Simple Stochastic Model of Point Source Solute Transport in Rivers Based on Gauging Station Data with Implications for Sampling Requirements. *Water Res.* **1999**, *33* (14), 3171–3181. https://doi.org/10.1016/S0043-1354(99)00026-3.

(10) Facchi, A.; Gandolfi, C.; Whelan, M. J. A Comparison of River Water Quality Sampling Methodologies under Highly Variable Load Conditions. *Chemosphere* **2007**, *66* (4), 746–756. https://doi.org/10.1016/j.chemosphere.2006.07.050.

(11) Ehalt Macedo, H.; Lehner, B.; Nicell, J.; Grill, G.; Li, J.; Limtong, A.; Shakya, R. Distribution and Characteristics of Wastewater Treatment Plants within the Global River Network. *Earth Syst. Sci. Data* **2022**, *14* (2), 559–577. https://doi.org/10.5194/essd-14-559-2022.

(12) Whelan, M. J.; Everitt, T.; Villa, R. A Mass Transfer Model of Ammonia Volatilisation from Anaerobic Digestate. *Waste Manag.* **2010**, *30* (10), 1808–1812. https://doi.org/10.1016/j.wasman.2009.08.012.

(13) Emerson, K.; Russo, R. C.; Lund, R. E.; Thurston, R. V. Aqueous Ammonia Equilibrium Calculations: Effect of pH and Temperature. *J. Fish. Res. Board Can.* **1975**, *32* (12), 2379–2383. https://doi.org/10.1139/f75-274.
